# Insights on the Emergence of *Mycobacterium tuberculosis* from the Analysis of *Mycobacterium kansasii*

**DOI:** 10.1093/gbe/evv035

**Published:** 2015-02-25

**Authors:** Joyce Wang, Fiona McIntosh, Nicolas Radomski, Ken Dewar, Roxane Simeone, Jost Enninga, Roland Brosch, Eduardo P. Rocha, Frédéric J. Veyrier, Marcel A. Behr

**Affiliations:** ^1^Department of Microbiology and Immunology, McGill University, Montreal, Québec, Canada; ^2^McGill International TB Centre, Montreal, Québec, Canada; ^3^Research Institute of the McGill University Health Centre, Montreal, Québec, Canada; ^4^McGill University and Génome Québec Innovation Center, Montreal, Québec, Canada; ^5^Unit for Integrated Mycobacterial Pathogenomics, Institut Pasteur, Paris, France; ^6^Dynamics of Host-Pathogen Interactions Unit, Institut Pasteur, Paris, France; ^7^Microbial Evolutionary Genomics Unit, Institut Pasteur, Paris, France; ^8^INRS-Institut Armand-Frappier, Laval, Québec, Canada; ^9^Department of Medicine, McGill University, Montreal, Québec, Canada

**Keywords:** mycobacteria, phylogeny, comparative genomics, virulence, *Mycobacterium kansasii*

## Abstract

By phylogenetic analysis, *Mycobacterium kansasii* is closely related to *Mycobacterium tuberculosis.* Yet, although both organisms cause pulmonary disease, *M. tuberculosis* is a global health menace, whereas *M. kansasii* is an opportunistic pathogen. To illuminate the differences between these organisms, we have sequenced the genome of *M. kansasii* ATCC 12478 and its plasmid (pMK12478) and conducted side-by-side in vitro and in vivo investigations of these two organisms. The *M. kansasii* genome is 6,432,277 bp, more than 2 Mb longer than that of *M. tuberculosis* H37Rv, and the plasmid contains 144,951 bp. Pairwise comparisons reveal conserved and discordant genes and genomic regions. A notable example of genomic conservation is the virulence locus ESX-1, which is intact and functional in the low-virulence *M. kansasii,* potentially mediating phagosomal disruption. Differences between these organisms include a decreased predicted metabolic capacity, an increased proportion of toxin–antitoxin genes, and the acquisition of *M. tuberculosis*-specific genes in the pathogen since their common ancestor. Consistent with their distinct epidemiologic profiles, following infection of C57BL/6 mice, *M. kansasii* counts increased by less than 10-fold over 6 weeks, whereas *M. tuberculosis* counts increased by over 10,000-fold in just 3 weeks. Together, these data suggest that *M. kansasii *can serve as an image of the environmental ancestor of *M. tuberculosis *before its emergence as a professional pathogen, and can be used as a model organism to study the switch from an environmental opportunistic pathogen to a professional host-restricted pathogen.

## Introduction

First identified in 1953 as the “yellow bacillus” (Pollak and Buhler 1953), *Mycobacterium kansasii* is an acid-fast bacterium that can cause a pulmonary disease in immunocompromised individuals and those with underlying pulmonary conditions such as chronic obstructive pulmonary disease and silicosis (Lillo et al. 1990; Corbett et al. 1999; Canueto-Quintero et al. 2003; Griffith et al. 2007). This disease resembles that caused by *Mycobacterium tuberculosis* in that patients experience similar symptoms (chest pain, productive cough, and weight loss), with comparable radiographic features (Evans, Colville, et al. 1996; Evans, Crisp, et al. 1996; Ellis 2004) and both infections can be treated with standard antituberculosis agents (Griffith et al. 2007). However, although tuberculosis (TB) is a global pandemic (World Health Organization 2013), *M. kansasii* infections are uncommon in the general population (Good and Snider 1982; Marras et al. 2007; Cassidy et al. 2009), and human-to-human *M. kansasii *transmission, if any, has rarely been documented (Davies 1994; Ricketts et al. 2014). The absence of transmission of *M. kansasii *infection in humans marks an evolutionary dead-end for this environmental organism, a scenario also described in *Legionella,* an accidental pathogen, and animal–human zoonotic pathogens such as *Campylobacter jejuni *and *Salmonella enterica* (Sokurenko et al. 2006; Sanchez-Buso et al. 2014).

Unlike *M. kansasii*, which is frequently found in aquatic environments (McSwiggan and Collins 1974; Joynson 1979; Kaustova et al. 1981; Thomson et al. 2013), the tubercle bacillus *M. tuberculosis* has no identified environmental reservoir. Rather, both phylogeographic and paleo-DNA studies present *M. tuberculosis* as a human-adapted pathogen that originated in Africa and accompanied the migrations of modern humans throughout the world (Wirth et al. 2008; Gagneux 2012; Comas et al. 2013). Despite these clear epidemiologic differences, the organisms share many similarities. Similar to *M. tuberculosis*, *M. kansasii* can grow at 37 °C with growth seen after 2–3 weeks on Löwenstein–Jensen medium (Roberts 1981). *Mycobacterium kansasii *is also positive for urease production, thiophene-2-carboxylic hydrazide resistance, and nitrate reduction (Roberts 1981; Tsukamura 1985), phenocopying biochemical characteristics long used for the laboratory identification of *M. tuberculosis*. In contrast, *M. kansasii* is a photochromogenic bacterium that produces carotenoid pigments against UV damage, a feature common to environmental organisms but lacking in *M. tuberculosis* (Tsukamura 1964; Robledo et al. 2011). Additionally, *M. kansasii* can utilize a much wider array of carbon and nitrogen sources to support growth than *M. tuberculosis* (Tsukamura et al. 1969; Tsukamura 1983), potentially capitalizing on a broader pool of nutrient sources in the environment.

As more complete mycobacterial genomes become available, comparative genomic studies provide the opportunity to identify testable differences between related, but biologically distinct species. For example, the smooth tubercle bacilli, exemplified by *Mycobacterium canettii*, are considered to be the most closely related species to *M. tuberculosis,* yet a number of genetic differences have been documented between these organisms (Gutierrez et al. 2005; Fabre et al. 2010; Supply et al. 2013). The observation that *M. canettii* causes TB in apparently immunocompetent hosts, along with its lack of a known environmental reservoir, together suggests that the biology of *M. canettii* may be more similar to *M. tuberculosis* than their putative environmental ancestor. To elucidate the speciation of *M. tuberculosis *on a larger evolutionary scale, we have compared *M. tuberculosis *with *M. kansasii*. Previous analysis, using multilocus sequence analysis, has identified *M. kansasii* as the environmental mycobacterium most closely related to the *M. tuberculosis* complex organisms (Veyrier et al. 2009). In this study, we determined the genome of *M. kansasii* and conducted comprehensive comparative genomic analyses between *M. kansasii* and *M. tuberculosis*. By apposing their genomes with phenotypic characterization, we present a novel perspective on the emergence of *M. tuberculosis* as a “professional” pathogen.

## Materials and Methods

### *Mycobacterium kansasii *and *M. tuberculosis *Strains

*Mycobacterium kansasii* ATCC 12478 (Hauduroy) is a clinical isolate now a type strain; belongs to subtype I (Richter et al. 1999). *Mycobacterium tuberculosis *H37Rv is a well-characterized virulent laboratory strain; *M. tuberculosis*:ΔRD1 and *M. tuberculosis*:Δ*esat-6* were gifts from Dr David Sherman (University of Washington). *Mycobacterium kansasii* clinical strains (62359, 62759, 62349, and 63327) were collected from the McGill University Health Centre between 2006 and 2010.

### Genome Sequencing and Annotation

The first draft of *M. kansasii *genome was assembled following shotgun sequencing using the Genome Sequencer with 33 × coverage (Veyrier et al. 2009). In this study, we prepared ultrapure, intact genomic DNA for the Pacific Biosciences (PacBio) sequencing platform to generate a gapless genome. DNA was extracted using the PowerSoil DNA isolation kit (MoBio, Carlsbad, CA, USA). Library preparation and sequencing reaction were carried out as described previously at Genome Quebec (Labrie et al. 2014). The sequenced genome was assembled using HGAP. Genome annotation was performed by the National Center for Biotechnology Information (NCBI) Prokaryotic Genome Annotation Pipeline using GeneMarkS (Besemer et al. 2001); features annotated include coding sequences (CDS), ribosomal RNA (rRNA), transfer RNA (tRNA), noncoding RNA (ncRNA), and repeat regions. Additional analysis was carried out by Rapid Annotation by Subsystems Technology (RAST) to organize genes into functional groups or pathways (Aziz et al. 2008).

### Strain Subtyping and Plasmid Detection

*Mycobacterium kansasii* ATCC 12478 and four clinical strains were subtyped using polymerase chain reaction (PCR)-restriction fragment length polymorphism and 16S–23S ITS sequencing as described in Alcaide et al. (1997) and Richter et al. (1999). The presence of plasmid was detected by PCR. Standard PCR reaction was carried out with an annealing temperature of 55 °C with forward (CTACCGCGACTACAACACCA) and reverse (GGGGTGAACGTGAGGTCATA) primers amplifying a 213-bp region in the conjugative transfer relaxase gene (*MKAN_29815*) found to be conserved in mycobacterial plasmids (Ummels et al. 2014). 

### Amino Acid Sequence-Based Phylogeny

The core genome was built by identifying proteins that were bidirectional best hits between pairs of genomes with at least 40% identity in protein sequence and less than 20% difference in size (Vieira-Silva et al. 2011). The intersection of the pairwise list of bidirectional best-hits provided the core genome of the clade. Each family of the core genome was then aligned using the protein sequence with MUSCLE 3.52 (Edgar 2004). The software BMGE was used to identify and extract suitable regions from multiple alignments using the BLOSUM62 matrix (Criscuolo and Gribaldo 2010). The resulting alignment had 91,530 distinct alignment patterns and was used to build a phylogenetic tree of the clade with RaxML 7.2.7 using the model PROTGAMMALG, with 100 bootstrap replicates (Stamatakis 2014). The resulting tree was visualized with FigTree (http://tree.bio.ed.ac.uk/software/figtree/, last accessed March 4, 2015).

Genomes used in this analysis included: *M. abscessus*, *M. abscessus* subspecies *bolletii*, *M. *sp. MCS, *M. *sp. KMS, *M. *sp. JLS, *Mycobacterium gilvum *Spyr1, *M. gilvum* PYR-GCK, *Mycobacterium vanbaalenii *PYR-1, *Mycobacterium vaccae*, *Mycobacterium chubuense, Mycobacterium phlei*, *Mycobacterium hassiacum*, *Mycobacterium rhodesiae* NBB3, *Mycobacterium tusciae*, *Mycobacterium mageritense*, *Mycobacterium fortuitum*, *Mycobacterium smegmatis *MC^2^155, *Mycobacterium neoaurum *VKM Ac-1815D, *Mycobacterium thermoresistable*, *M. *sp. JDM601, *Mycobacterium xenopi*, *Mycobacterium indicus pranii *MTCC 9506, *Mycobacterium intracellulare *MOTT-64, *M. intracellulare *ATCC 13950, *Mycobacterium yongonense *05-1390, *M. *sp. MOTT36Y, *Mycobacterium colombiense*, *Mycobacterium avium *104, *M. avium *sp. *paratuberculosis *K-10, *Mycobacterium parascrofulaceum*, *Mycobacterium leprae *TN, *M. kansasii *ATCC 12478, *Mycobacterium gastri*, *Mycobacterium liflandii *128FXT, *Mycobacterium ulcerans *Agy99, *Mycobacterium marinum *M, *M. canettii *CIPT 140010059, *M. tuberculosis *H37Rv, *Mycobacterium bovis *AF2122/97*, *and *Mycobacterium africanum* GM041182*.*

Related species in the same order (*Actinomycetales*) were used as an outgroup; RHEQ: *Rhodococcus equi*; NOFA, *Nocardia farcinica*.

### Genome Comparison between *M. kansasii* and *M. tuberculosis*

Genes present in *M. tuberculosis* but absent in other mycobacteria, including *M. kansasii, *have been described (Veyrier et al. 2009). To generate the list of genes potentially have been lost in *M. tuberculosis *since its divergence from other mycobacteria, the CDS of *M. tuberculosis* were compared with other mycobacteria including *M. abscessus*, *M. gilvum*, *M. intracellulare*, *M. *sp. JLS*, M. vanbaalenii*, *M. smegmatis*, *M. *sp. KMS, *M. *sp. MCS, *M. avium* 104, *M. avium *subsp. *paratuberculosis*, *M. ulcerans*, and *M. kansasii*, using criteria described in Veyrier et al. (2009). The resulting gene list was categorized into Clusters of Orthologous Groups (COG) functional categories (Tatusov et al. 2000).

### In Vitro Comparisons

The growth of *M. tuberculosis* H37Rv and *M. kansasii *in 7H9 supplemented with ADC enrichment and 0.2% Tween-80 at 37 °C with rolling was monitored with a spectrophotometer at OD_600_ in triplicates every 24 h for 4 days. Bacteria were also streaked out on 7H10 agar plates for morphological observation. To verify the presence of a functional ESX-1 secretion system, culture supernatants for *M. tuberculosis*, *M. tuberculosis*:ΔRD1, *M. tuberculosis*:Δ*esat-6*, and *M. kansasii* were prepared according to Chen et al. (2012) and probed with 1:5,000 monoclonal anti-ESAT-6 antibody (11G4, Abcam). The abundance of each protein was measured and quantified using ImageJ (Schneider et al. 2012).

### Phagosomal Perturbation Assay

Detection of phagosomal rupture was performed as described using a single-cell fluorescence resonance energy transfer (FRET)-based assay (Ray et al. 2010; Simeone et al. 2012). Briefly, *M. tuberculosis*, *M. tuberculosis*:ΔRD1, and *M. kansasii *were used to infect phorbol-myristate-acetate (PMA)-differentiated THP-1 macrophages. THP-1 cells were incubated with CCF-4-AM (Invitrogen), an esterified, lipophilic form of the CCF-4 substrate that can readily enter into cells, where it is converted by endogenous cytoplasmic esterases into CCF-4 that is negatively charged. CCF-4 is retained in the cytosol and emits green fluorescence (535 nm) upon stimulation at 405 nm, due to FRET from the 7-hydroxycoumarin donor to the fluoroscein acceptor. When in contact with endogenous beta-lactamases produced by mycobacteria, as occurs when the phagosomal compartment is disrupted, CCF-4 is cleaved, emitting at 450 nm when excited at 405 nm. All experimental steps were performed in the presence of probenecid upon CCF-4-AM treatment to avoid the export of the fluorescent reporter. The 450/535 nm intensity ratio measurements were obtained and analyzed by algorithms provided by the Metamorph software, as previously reported (Ray et al. 2010).

### In Vivo Comparisons

Eight-week-old female C57BL/6 mice purchased from Jackson Laboratories were aerosol-infected with wild-type *M. tuberculosis*, *M. tuberculosis*:ΔRD1, and *M. kansasii*, as previously described (Hansen et al. 2014). Five mice from each group were sacrificed 1 day postinfection and their lung homogenates were plated out for the enumeration of bacteria. At days 21 and 42 postinfection, the lungs and spleens of infected mice were homogenized for bacterial quantification by serial dilution. All animal experiments were in accordance with the regulations of the Canadian Council of Animal Care and approved by the McGill University Animal Committee.

## Results

### *Mycobacterium kansasii* Is among the Closest Species to *M. tuberculosis*

**Fig. 1.—:**
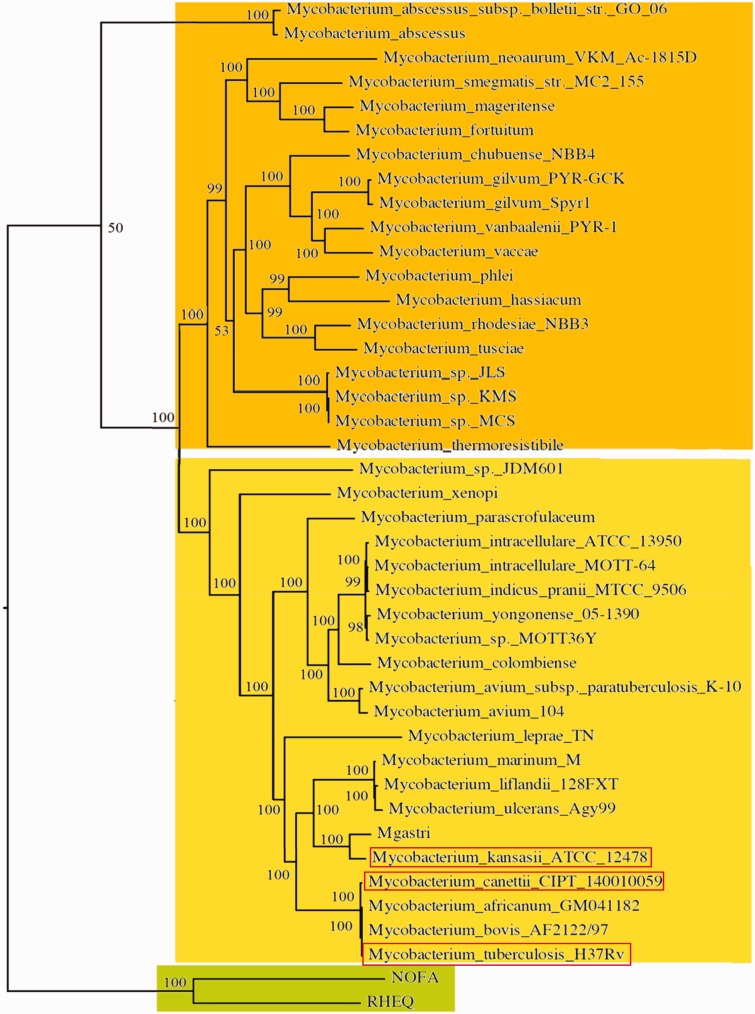
Phylogenetic relationships among *Mycobacterium* genus. The phylogenetic tree was generated using RAxML. The scale bar represents amino acid changes per site. Rapid growing species are shadowed in orange, slow growing species are shadowed in yellow, and outgroup species are shadowed in green. *Mycobacterium tuberculosis*, *M. canettii*, and *M. kansasii *are boxed in red.

We identified the proteins conserved across 40 available mycobacterial genomes and two outgroups (*R. equi *and *N. farcinica*). We then used this information to build a rooted phylogenetic tree of the *Mycobacterium *genus. This tree can be divided into three groups: The outgroup actinobacteria (*R. equi* and *N. farcinica)*, the rapid-growing mycobacteria (such as *M. smegmatis*), and emerging from this group, the slow-growing mycobacteria. In this slow-growing sublineage, members of the *M. tuberculosis *complex (*M. tuberculosis, M. bovis*, and *M. africanum*) clustered together, next to the early branching tubercle bacilli of the *M. canettii *clade (Fabre et al. 2004; Supply et al. 2013). The next most closely related organisms include *M. kansasii* and *M. marinum *(fig. 1), the latter a pathogenic species that can cause skin disease and lymphangitis in humans, and grows optimally at 30 °C.

### The *M. kansasii* Genome

**Fig. 2.—:**
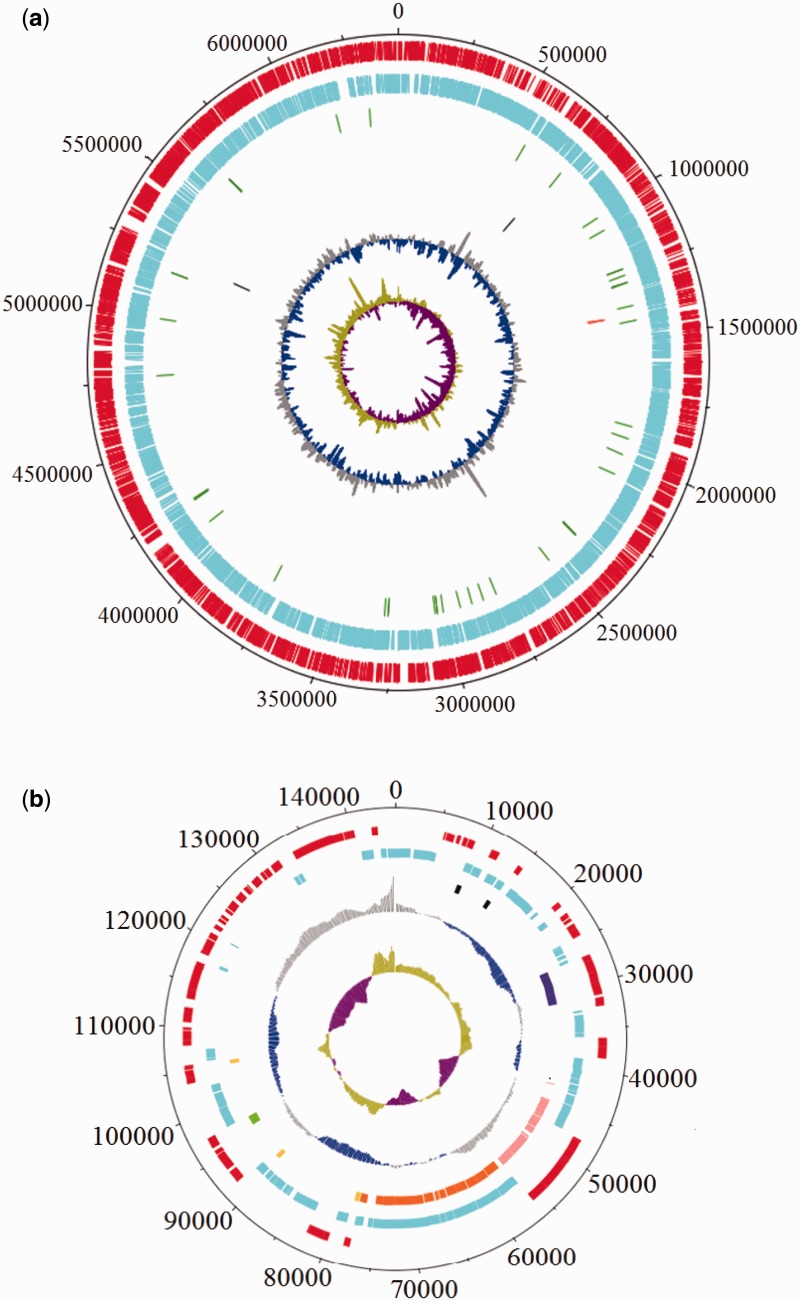
(*A*) Circular representation and annotation features of *M. kansasii* ATCC 12478 chromosome. The two outermost circles represent forward and reverse-strand CDS (red and blue, respectively). tRNA is shown in green, rRNA in orange, and ncRNA in black. The two innermost blue/gray and olive/purple circles represent G + C content and GC skew, respectively. (*B*) Circular representation of the pMK12478 plasmid. Forward and reverse CDS and G + C content and GC skew are labeled in the same color scheme as the chromosome. Genes coding for putative sigma factors are coded in black; MCE in dark purple; replication protein in green; toxin–antitoxin in yellow, putative T4SS in pink; putative T7SS in orange (NCBI annotation; Ummels et al. 2014).

The *M. kansasii* ATCC 12478 genome consists of a chromosome of 6,432,277 bp (NCBI Reference Sequence NC_022663.1) plus a plasmid, pMK12478, which is 144,951 bp in size (NCBI Reference Sequence: NC_022654.1). The circular representation of the genome was generated using DNAplotter (Carver et al. 2009) and is shown in figure 2*A*. The annotated genome indicated the existence of 5,712 CDS in the chromosome, 3 rRNAs (5S, 16S, and 23S), 46 tRNAs, and 2 ncRNAs. Additionally, there were 10 disrupted pseudogenes, previously encoding 2 membrane proteins, 1 dihydrodipicolinate reductase, 1 serine/threonine protein kinase, 1 imidazole glycerol phosphate synthase, 1 transposase, 1 integrase, and 3 hypothetical proteins.

**Fig. 3.—:**
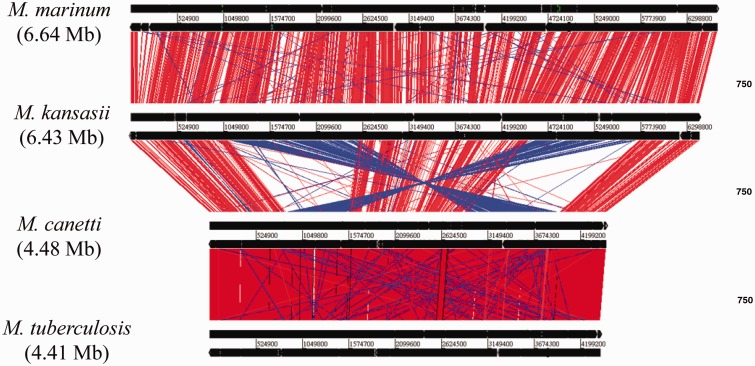
Comparisons of the genomes of *M. tuberculosis*, *M. canettii*, *M. kansasii*, and *M. marinum. *Red lines indicate local colinear blocks of DNA–DNA similarity and blue lines indicate rearranged regions.

The comparison of the genome organization of *M. tuberculosis*, *M. canettii *CIPT 140010059, *M. kansasii,* and *M. marinum *was visualized by Artemis Comparison Tool (Carver et al. 2005). As shown in figure 3, two large chromosomal inversions separate the group of *M. canettii* and *M. tuberculosis *from that of *M. marinum* and *M. kansasii*. *Mycobacterium kansasii* and *M. marinum *genomes display large regions of similarity whereas *M. canettii* and *M. tuberculosis *exhibit high synteny conservation, consistent with their phylogenetic relationships. Individual genome comparisons between *M. marinum*, *M. kansasii,* and *M. tuberculosis*/*M. canettii* are presented in supplementary figure S1, Supplementary Material online.

The *M. kansasii* plasmid, pMK12478, is predicted to encode 154 proteins, including 4 mammalian cell entry (MCE) proteins, 1 PE family protein, several secretion-associated proteins, toxin–antitoxin systems, as well as transcriptional regulators and sigma factors (fig. 2*B*). *Mycobacterium kansasii* ATCC 12478 belongs to the subgroup 1, a subgroup strongly associated with diseases (Taillard et al. 2003). To assess whether the plasmid is associated with pathogenicity, we tested four recent clinical isolates from the McGill University Health Centre mycobacterial laboratory. Although all isolates were from subgroup 1, in accordance with previous report (Taillard et al. 2003), two of these isolates did not contain the plasmid, suggesting that the plasmid is dispensable for clinical disease in humans.

### Changes in Genetic Content during the Step-Wise Emergence of *M. tuberculosis*

**Fig. 4.—:**
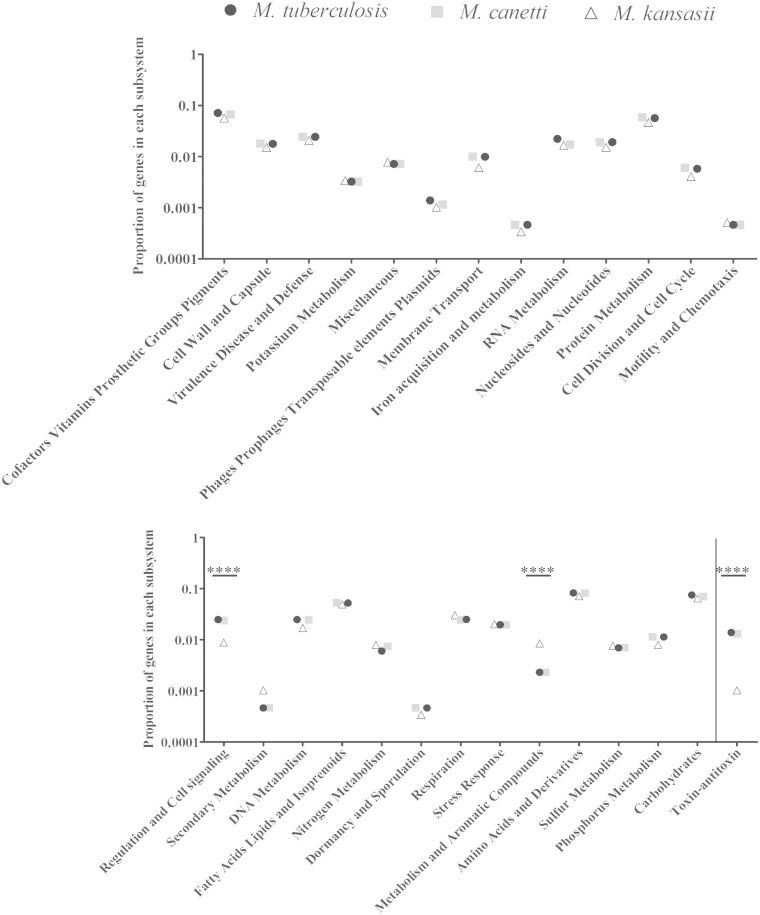
The proportion of genes in each subsystem in *M. tuberculosis*, *M. canettii*, and *M. kansasii,* Asterisks indicate the results of the statistical tests comparing *M. tuberculosis* and *M. kansasii*. Sample proportions were compared using a two-sample *Z*-test. ****P < 0.0001.

To compare the proportion of genes in different functional metabolic categories, RAST was used (Aziz et al. 2008), and visualized by SEED Viewer (Overbeek et al. 2014). RAST estimated 5,888 CDS in the genome. Of these, 1,953 were assigned into 27 subsystems, a collection of functional roles that constitute a certain biological pathway or structural complex, providing predictions on just over one-third of the genes (1,953/5,888 = 34%) (Overbeek et al. 2005; Meyer et al. 2009). Applying the same methodology to the smaller genome of *M. tuberculosis *H37Rv (Cole et al. 1998), there was a lower number, but higher proportion, of predicted CDS assigned to subsystems (1,619/4,317 = 38%). Differences in proportions of genes in each subsystem were assessed using the two-sample *Z*-test (two-tailed). A Bonferroni correction was used to account for multiple testing; therefore, a result with *P* < 0.05/27, or *P* < 0.0019 was considered significant at an α of 0.05. Compared with *M. kansasii*, *M. tuberculosis *has a greater proportion of its genome devoted to regulation and cell signaling. A closer inspection of genes in this category revealed that this difference is mostly due to the large number of genes involved in toxin–antitoxin systems (*P* < 0.0001) (fig. 4 and supplementary table S1, Supplementary Material online). To evaluate whether these differences were specific to the comparison between the two genomes, or representative of a larger dichotomy between *M. tuberculosis* and nontuberculous mycobacteria (NTM), we examined the genomes of the slow-growing *M. marinum*, *M. avium *subsp. *hominissuis*, *M. intracellulare*, *M. avium *subsp. *paratuberculosis*, and the fast-growing *M. abscessus*, *M. vanbaalenii*, *M. gilvum*, and *M. smegmatis. *Compared with these genomes, *M. tuberculosis* still shows a higher proportion of genes in the regulation and cell signaling subsystem, again driven by the number of toxin–antitoxin genes, whereas all other mycobacteria devote much more of their genomes to metabolism of various compounds. We also noted that the proportion of genes in all subsystems examined was the same in *M. tuberculosis *and *M. canettii *(*P* > 0.1) (supplementary table S1 and fig. S2, Supplementary Material online).

To assess which *M. kansasii* genes absent from *M. tuberculosis* were most likely deleted during the emergence of *M. tuberculosis*, we identified 349 genes present in *M. kansasii *and 13 other nontuberculous mycobacteria that are exclusively absent from *M. tuberculosis *(346 out of these 349 genes are also lost in *M. canettii* CIPT 140010059) (supplementary table S2, Supplementary Material online). Based on their COG functional categories, many of these genes are predicted to be involved in energy production and conversion (*n* = 34), lipid metabolism (*n* = 46), and biosynthesis, transport, and catabolism of secondary metabolites (*n* = 37), consistent with the previously published observation of a greater metabolic capacity of *M. kansasii* (Tsukamura et al. 1969; Tsukamura 1983)*.*

### Comparison of Virulence Determinants of *M. tuberculosis*

**Fig. 5.—:**
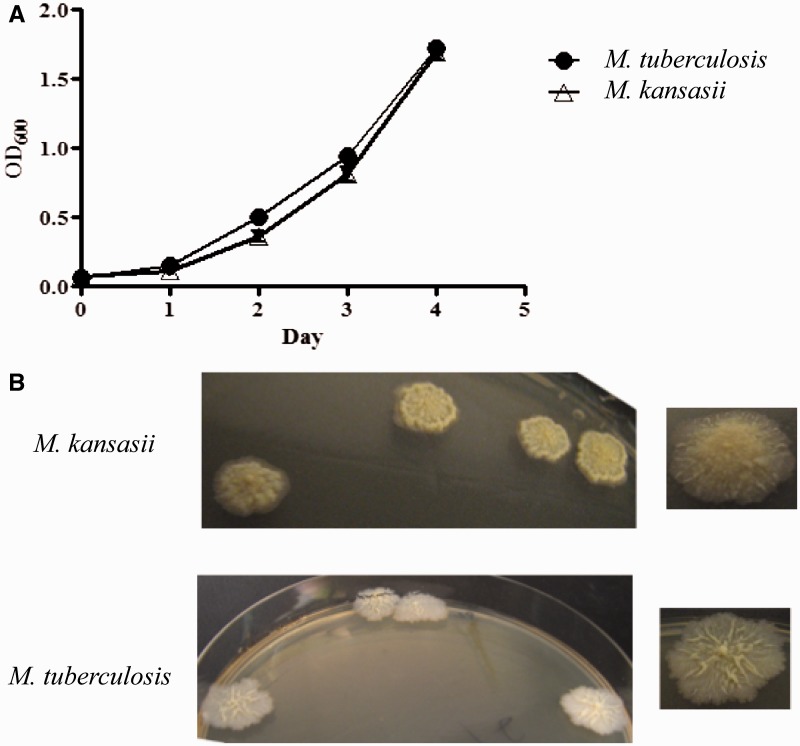
(*A*) Comparative growth of *M. kansasii* and *M. tuberculosis* in 7H9 at 37 °C. (*B*) Morphological characteristics of *M. kansasii *(top panel) and *M. tuberculosis* (bottom panel) on 7H10 agar.

A growing number of *M. tuberculosis* genes have been experimentally shown to be necessary for full virulence of this organism, so we investigated whether any of these was absent from the genome of *M. kansasii*. We found that the two-component signal transduction system PhoPR and dormancy regulon DosR/S/T are conserved in the low-virulence *M. kansasii *(summarized in table 1) (Perez et al. 2001; Ryndak et al. 2008; Converse et al. 2009; Leistikow et al. 2010). Based on RAST annotation, the *M. tuberculosis *genome has 87 PE and 66 PPE proteins, implicated in pathogen–host interaction (Sampson 2011); in comparison, the *M. kansasii *genome is predicted to encode 72 PE and 141 PPE proteins. *Mycobacterium tuberculosis *has 36 *mce-*associated genes (Zhang and Xie 2011) and we found a similar number (36 + 1 on the plasmid) in *M. kansasii*. In addition, more than 65% of the antigens encoded by *M. tuberculosis* are also present in *M. kansasii* including MPT70, MPT60, TB8.4, antigen 85, ESAT-6, and CFP-10, with greater than 75% amino acid homology and 80% coverage (Comas et al. 2010). Finally, in *M. tuberculosis*, the ESX-1 locus has been shown to be crucial for full virulence (Brodin et al. 2006; MacGurn and Cox 2007), with a variety of phenotypes shown to depend upon the presence of an intact ESX-1 locus (Abdallah et al. 2007). At the genomic level *M. kansasii* has an intact *ESX-1* locus encoding building blocks of the Type VII secretion system (T7SS) (*MKAN_14470–MKAN_14850*), as well as the coregulated *espA–espC–espD* gene cluster (*MKAN_12680, 12685, 12690*), with protein similarities ranging from 58% to 95% (fig. 5*B*). Therefore, *M. kansasii* encodes orthologs for all these recognized virulence determinants of *M. tuberculosis*. Notably, it was noted that the putative ortholog of the *espG_1_* (*Rv3866*) gene of *M. tuberculosis, MKAN_07615* is encoded outside the ESX-1 locus. As the *espG*-gene products were suggested to represent potential chaperon proteins that interact with ESX-system-specific PE/PPE proteins and guide specific T7SS effectors to their “correct” ESX secretion system, this observation is of potential interest (Daleke et al. 2012).

**Table 1:** Summary of Shared and Discordant Genomic and Phenotypic Features of the *Mycobacterium kansasii *and *Mycobacterium tuberculosis*

	*M. tuberculosis*	*M. kansasii*
Disease	Host-adapted	Opportunistic
Transmission	Human–human	Water–human
Reservoir	Human	Environment
Genome size	4.4 Mb	6.4 Mb
Protein genes	4,018	5,866
% GC content	66	66
Presence of PhoPR 2-component system	Yes (*Rv0757/8*)	Yes (*Mkan_11335/30*)
Presence of DosR/S/T regulon	Yes (*Rv3133c/3132c/2027c*)	Yes (*Mkan_22405/22410/22410*)
# PE/PPE proteins	153	213
# *mce-*associated genes	36	36
Proportion of metabolism genes	0.002316423	0.0084918
Proportion of toxin/antitoxin genes	0.013899	0.001019
Presence of ESX-1	Yes	Yes
T-cell antigens	77	53
Phagosomal disruption	Yes	Yes
Replication in mouse lungs	4.5 log	0.5 log

Note.—The number of PE/PPE proteins and *mce-*associated genes are based on RAST annotation.

### Changes in the In Vitro Phenotype during the Step-Wise Emergence of *M. tuberculosis*

The growth patterns and morphologies of *M. kansasii *and *M. tuberculosis* are shown in figure 5*. *In 7H9 broth with rolling, *M. kansasii *and *M. tuberculosis* grew at similar rates with doubling times of 19.6 and 20.9 h, respectively (fig. 5*A*). After growth on 7H10 agar for 8–10 weeks at 37 °C, *M. kansasii* colonies appeared slightly raised, smooth, with a light yellow color whereas *M. tuberculosis *colonies were more textured, wrinkled with no pigmentation (fig. 5*B*). Although the chemical basis for the rough morphology has not been elucidated in *M. tuberculosis *(Lemassu et al. 1992), it has been reported that *M. kansasii* strains, such as ATCC 12478 (also designated as TMC 1204) that contain acyltrehalose-containing lipooligosaccharides, exhibit a smooth morphology (Belisle and Brennan 1989).

**Fig. 6.—:**
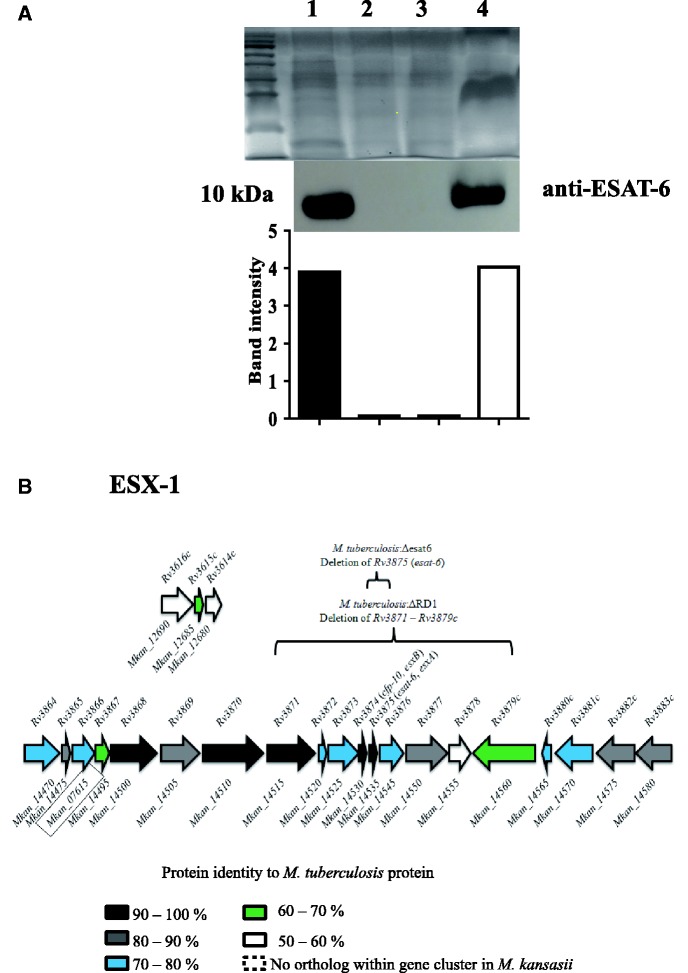
(*A*) Top panel: SDS-PAGE analysis of mycobacterial culture filtrate proteins; lane 1: *M. tuberculosis*; lane 2: *M. tuberculosis:*ΔRD1; lane 3: *M. tuberculosis:Δesat-6* ; lane 4: *M. kansasii*. Middle panel: Western blot showing the presence of ESAT-6 in *M. tuberculosis* and *M. kansasii* culture filtrates but not *M. tuberculosis:*ΔRD1 or *M. tuberculosis:Δesat-6*. Bottom panel: Band intensities measured by ImageJ and represented in histogram. (*B*) Schematic illustration of the organization of ESX-1 and orthologous genes in *M. kansasii*. Each gene is color-coded according to the degree of protein similarity when aligning *M. kansasii *amino acid sequence to that of *M. *tuberculosis. *Mycobacterium kansasii* genes that are orthologous to *M. tuberculosis *ESX genes but not organized in a gene cluster are boxed. Regions deleted in *M. tuberculosis:*ΔRD1 and *M. tuberculosis:Δesat-6 *are also indicated.

To verify that the ESX-1 system is functionally intact in *M. kansasii* ATCC 12478, we conducted immunoblotting for the 6-kDa early secreted antigenic target ESAT-6 protein, one of the most abundant T7SS-associated proteins (Sorensen et al. 1995; Harboe et al. 1996). As expected, we detected ESAT-6 in the culture filtrate in both species, but not *M. tuberculosis*:ΔRD1 (region of difference containing the *esat-6* gene as well as several other indispensable components of the T7SS [Pym et al. 2002; Guinn et al. 2004]), or *M. tuberculosis*:Δ*esat-6* (fig. 6*A* and *B*).

**Fig. 7.—:**
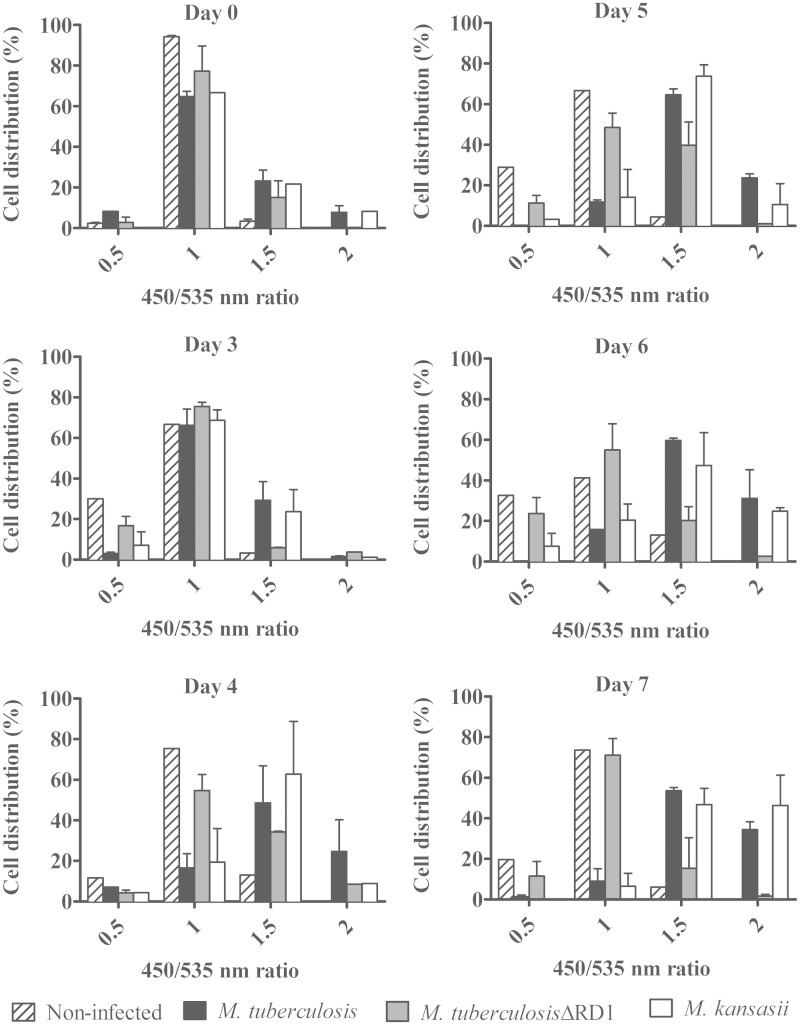
*Mycobacterium tuberculosis* and *M. kansasii* induced a change in fluorescence ratio starting on day 4 postinfection whereas *M. tuberculosis*:1 did not show such activity.

ESAT-6 is required for *M. tuberculosis* to access the cytosol after phagocytosis (van der Wel et al. 2007; Houben et al. 2012; Simeone et al. 2012). The production and secretion of ESAT-6 in *M. kansasii* led us to test whether this species is also capable of compromising the phagosomal membrane inside the macrophage using a previously established FRET reporter assay (Ray et al. 2010; Simeone et al. 2012). Indeed, as shown in figure 7, over the course of a 7-day infection, macrophages containing the FRET reporter infected with *M. tuberculosis *and *M. kansasii* began to exhibit a decrease in FRET signal in the cytosol from 535 to 450 nm with time plotted as fluorescent ratios of the two measured channels, suggesting that the integrity of the phagosomal membrane was compromised. This phenotype was not observed in macrophages infected with *M. tuberculosis*:ΔRD1, as has been previously published (van der Wel et al. 2007), indicating that the behavior of *M. kansasii* within a macrophage parallels that of fully virulent *M. tuberculosis*, extending previous observations with *M. kansasii* type I strains (Houben et al. 2012).

### Changes in the In Vivo Phenotype during the Step-Wise Emergence of *M. tuberculosis*

**Fig. 8.—:**
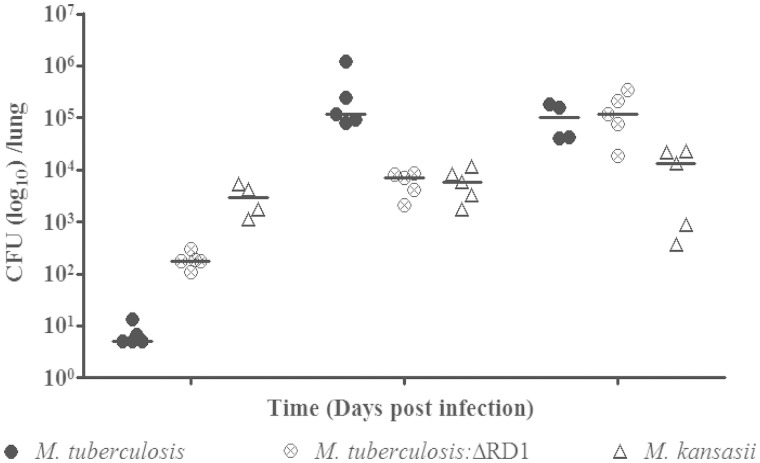
Bacterial burden in the lungs of mice infected through an aerosol route with *M. tuberculosis*, *M. tuberculosis:*ΔRD1, and *M. kansasii* over the course of 42 days.

In preliminary experiments with *M*. *kansasii*, infection of C57BL/6 mice with the low dose of bacteria used for *M. tuberculosis* (∼50–100 colony forming units) did not result in a consistently detectable infection at 3 weeks after aerosolization, suggesting either a failure to deliver the organism to the airways or the rapid elimination of *M. kansasii* upon infection. However, assessment at 1 and 24 h postinfection with a higher inoculum showed comparable bacterial loads in mouse lungs, arguing against rapid elimination after exposure (supplementary table S3, Supplementary Material online). Therefore, to monitor and quantify *M. kansasii* burden over time, we used a higher infectious dose than we use for *M. tuberculosis*. As shown in figure 8, following aerosol infection with *M. kansasii*, there was less than 10-fold difference in bacterial burden from day 1 through day 42 of infection. In contrast, for *M. tuberculosis* we observed over 10,000-fold increase in bacterial numbers by day 21. To contrast the phenotype of ESX-1-containing *M. kansasii* with an ESX-1 mutant of *M. tuberculosis*, we infected a third group of mice with an intermediate dose of *M. tuberculosis*:ΔRD1. In the first 21 days, the *M. tuberculosis* mutant had increased in burden by 100-fold, and by day 42, it had reached the same bacterial burden as wild-type *M. tuberculosis*, as has been previously described (Lewis et al. 2003). Thus, *M. kansasii*, with an intact ESX-1 locus manifest orders of magnitude reduced in vivo fitness compared with both wild-type *M. tuberculosis* and the ESX-1 mutant of *M. tuberculosis* following experimental aerosol infection in mice.

## Discussion

In this study, we analyzed the complete genome sequence of *M. kansasii*, an opportunistic pathogen that causes a sporadic, nontransmissible TB-like disease in humans. In agreement with a previous report, *M. kansasii* is one of the phylogenetically closest species to *M. tuberculosis*, an observation supported by the two genomes sharing extended regions of collinearity and synteny. The similarity between these genomes affords an opportunity to infer the common ancestor of these organisms and thereby suggest a sequence of events that resulted in the emergence from an environmental mycobacterium of a host-adapted professional pathogen.

Using the *M. kansasii* genome as a representative of a slow-growing environmental mycobacterium, the major genetic events that characterize the emergence of *M. tuberculosis* comparison are gene loss, gene acquisition, and gene amplification. These same processes are inferred when comparing *M. tuberculosis* to other nontuberculous mycobacteria, such as *M. marinum*, suggesting that much of the excess genetic material found in environmental mycobacteria codes for functions not required to survive in, and transmit between, mammalian hosts. Although it is possible that gene loss was selected because of the deletion of unnecessary functions, it also remains possible that certain antivirulence systems were present in environmental mycobacteria, such that their deletion actively contributed to enhanced virulence. As one putative example, the loss of the *cobF* gene during the evolution toward the MTBC should be mentioned. The gene *cobF*, which is absent from all MTBC members, is part of a gene cluster that is conserved in many environmental mycobacteria, including *M. kansasii* (*MKAN_09045*), and was also found present in all tested *M. canettii* strains (Supply et al. 2013). It remains to be determined whether the loss of this gene, whose gene product is predicted to be involved in cobalamin synthesis and endogenous vitamin B12 production (Gopinath et al. 2013), might have resulted in MTBC strains that depended upon the host for host-supplied substrates (Boritsch et al. 2014).

A complementary possibility to explain the enhanced virulence of *M. tuberculosis* involves that acquisition, through horizontal gene transfer, of novel *M. tuberculosis*-specific genes. The observation that many of these genes, including *Rv3377-8c *and *Rv0987/88*, are also found in *M. canettii*, which is considerably more virulent than *M. kansasii* in experimental infection models, supports the potential role of these acquired genes in the adaptation to a pathogenic lifestyle. A final possibility relates to the expansion of existing gene families. A previously published evolutionary analysis indicated that the considerable number of PE/PPE genes in *M. tuberculosis* is likely the result of gene expansion, rather than gene acquisition (Gey van Pittius et al. 2006). Our comparative genomic analysis detected a comparable amount of PE/PPE genes are seen between *M. kansasii* and *M. tuberculosis*, suggesting that this particular gene expansion did not coincide with the emergence of *M. tuberculosis*. Instead, we found that between these organisms there has been a markedly increased number of toxin–antitoxin genes in *M. tuberculosis*, as has been previously noted (Ramage et al. 2009). The apparent expansion of this gene family since their common ancestor argues for further investigation of the role toxin–antitoxin systems in the ability of *M. tuberculosis* to infect and cause disease.

Our genomic comparisons were also notable for the conservation of genes that have been experimentally implicated in TB pathogenesis. A nonexhaustive list of putative virulence factors of *M. tuberculosis* that were detected in *M. kansasii* is provided in table 1. Among these, one notes in the *M. kansasii* genome the presence of all five known *M. tuberculosis *ESX loci encoding the T7SS (fig. 6 and supplementary fig. S3, Supplementary Material online) (Bitter et al. 2009). Remarkably, the pMK12478 plasmid also contains a locus that putatively encodes a T7SS that is the most homologous to ESX-5 (Ummels et al. 2014) (supplementary fig. S3, Supplementary Material online). The presence of this plasmid carrying elements of T7SS and T4SS in *M. kansasii* adds new elements to the discussion on the origin and function of T7SS in mycobacteria. Beyond evaluating genomic conservation, we conducted functional studies of one of these ESX loci, named ESX-1, which has been implicated in a wide variety of *M. tuberculosis* virulence phenotypes. Previous studies have reported the presence of the ESX-1 secreted protein ESAT-6 in *M. kansasii* bacterial pellets as well as 3- to 5-week-old culture filtrates (Sorensen et al. 1995; Arend et al. 2005). Here, we showed that *M. kansasii* secreted similar amounts of ESAT-6 as *M. tuberculosis* after 4 days of growth in Sauton’s medium, a growth medium known to support ESAT-6 secretion (Champion 2013). As ESAT-6 has been shown to be necessary for *M. tuberculosis *to cause phagosomal perturbation, leading to bacterial relocalization to the cytosol (van der Wel et al. 2007; Simeone et al. 2012), we hypothesized that *M. kansasii* would likewise perturb the phagosomal membrane during macrophage infection. Like many other mycobacteria, *M. kansasii* produces endogenous beta-lactamase (Kwon et al. 1995), permitting us to use a FRET-based assay to detect whether *M. kansasii* could disrupt the phagosomal membrane, allowing the beta-lactamase to cleave the cytosolic FRET substrate and thereby change fluorescence emission upon excitation. Both *M. kansasii* and *M. tuberculosis *were able to disrupt the phagosomal membrane, which is likely a prerequisite for cytosol translocation as demonstrated by a *M. kansasii* clinical isolate (Houben et al. 2012). Thus, both genomic comparisons and intracellular infection studies point to key similarities between these organisms, begging the question of what additional virulence determinants have been acquired by *M. tuberculosis* since their common ancestor.

In experimental infections, *M. kansasii* showed considerably less growth in vivo, resulting in a persistent infection without clinical disease. These findings, along with the absence of a number of *M. tuberculosis-*specific genes, suggest that *M. kansasii* may lend itself to mechanistic studies through gain-of-function investigations involving the addition of *M. tuberculosis*-specific genes. Although *M. marinum* has been well utilized as a surrogate bacteria for a number of TB pathogenesis studies (Ramakrishnan 2012; Cambier et al. 2014), there are a number of differences between *M. marinum* and *M. tuberculosis*, including the faster growth rate of the former at a lower temperature, and the use of nonmammalian hosts. In contradistinction, *M. kansasii *grows in the same conditions, and at the same rate as *M. tuberculosis*, offering the possibility of a number of in vitro investigations, including transcriptomic, proteomic, and lipidomic comparisons. Additionally, *M. kansasii *can be used to generate productive pulmonary infection of standard mice used for experimental infection studies, albeit with lesser bacterial growth following infection, providing an in vivo phenotyping possibility to formally test the importance of in silico*-*derived differences between the organisms.

In conclusion, our comparative phenotypic and genomic study of *M. kansasii* and *M. tuberculosis *revealed that although these two species share numerous similarities, including the conservation of many *M. tuberculosis *virulence factors, they differ markedly in terms of in vivo fitness. These data support the search for novel *M. tuberculosis*-specific virulence factors not found in other mycobacteria, both for their fundamental value in understanding the pathogenesis of TB, but also for their potential translational value in species-specific diagnostic assays and novel immunization approaches.

## Supplementary Material

Supplementary tables S1–S3 and figures S1–S3 are available at *Genome Biology and Evolution* online (http://www.gbe.oxfordjournals.org/).

Supplementary Data
