# Supplementary material for: Insights on the Emergence of Mycobacterium tuberculosis from the Analysis of Mycobacterium kansasii
Source: Genome Biol Evol. 2015 Feb 25;7(3):856–70. doi: 10.1093/gbe/evv035 (PMC5322544; doi:10.1093/gbe/evv035)
Supplement: Supplementary Data [file supp_7_3_856__index.html]

Insights on the emergence of Mycobacterium tuberculosis from the analysis of Mycobacterium kansasii — Insights on the Emergence of Mycobacterium tuberculosis from the Analysis of Mycobacterium kansasii — Supplementary Data 

# Insights on the Emergence of *Mycobacterium tuberculosis* from the Analysis of *Mycobacterium kansasii*

## Supplementary Data

files

**Files in this Data Supplement:**

- Supplementary Data - xlsx file
